# The fiddler crab, *Minuca pugnax*, follows Bergmann's rule

**DOI:** 10.1002/ece3.5883

**Published:** 2019-12-09

**Authors:** David Samuel Johnson, Cynthia Crowley, Katherine Longmire, James Nelson, Bethany Williams, Serina Wittyngham

**Affiliations:** ^1^ Virginia Institute of Marine Science William & Mary Gloucester Point VA USA; ^2^ University of Vermont Burlington VT USA; ^3^ University of Louisiana Lafayette LA USA; ^4^ CSS, Inc. Fairfax VA USA

**Keywords:** Bergmann's rule, fiddler crab, Gulf of Maine, salt marsh, temperature–size rule, tropicalization

## Abstract

Bergmann's rule predicts that organisms at higher latitudes are larger than ones at lower latitudes. Here, we examine the body size pattern of the Atlantic marsh fiddler crab, *Minuca* *pugnax (formerly Uca pugnax)*, from salt marshes on the east coast of the United States across 12 degrees of latitude. We found that *M. pugnax* followed Bergmann's rule and that, on average, crab carapace width increased by 0.5 mm per degree of latitude. *Minuca pugnax* body size also followed the temperature–size rule with body size inversely related to mean water temperature. Because an organism's size influences its impact on an ecosystem, and *M. pugnax* is an ecosystem engineer that affects marsh functioning, the larger crabs at higher latitudes may have greater per‐capita impacts on salt marshes than the smaller crabs at lower latitudes.

## Graphical abstract

The fiddler crab, *Minuca pugnax*, follows Bergmann's rule, with the largest crabs at the highest latitudes. *M. pugnax* body size was negatively correlated with water temperature, following the temperature–size rule.

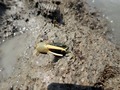

## 1 INTRODUCTION

One of the best‐known patterns in biogeography is Bergmann's rule. It predicts that organisms at higher latitudes are larger than ones at lower latitudes (Bergmann, 1847). We define Bergmann's rule as a biogeographic pattern, not a mechanism explaining the pattern (sensu Blackburn, Gaston, & Loder, 1999). Further, we define Bergmann's rule as applying to both inter‐ and intraspecific patterns of biogeography [intraspecific patterns are sometimes called James' rule (James, 1970), but we prefer to remain loyal to the original theorist]. Many organisms follow Bergmann's rule, including insects (Arnett & Gotelli, 2003; Ho, Pennings, & Carefoot, 2010), birds (Bergmann, 1847), snakes (Ashton, 2001), marine invertebrates (Ho et al., 2010; Jaramillo et al., 2017; Manyak‐Davis, Bell, & Sotka, 2013; Rosa, Gonzalez, Dierssen, & Seibel, 2012), and terrestrial and marine mammals (Ashton, Tracy, & Queiroz, 2000; Torres‐Romero, Morales‐Castilla, & Olalla‐Tarraga, 2016).

What drives Bergmann's rule? Bergmann (1847, based on translation by Salewski & Watt, 2017) originally hypothesized that the organisms he studied, birds, were larger in the colder, higher latitudes due to heat‐conservation (i.e., larger organisms have a lower surface area to volume ratio than smaller organisms and can therefore retain heat better; the heat‐conservation hypothesis). But the heat‐conservation hypothesis relies on internal regulation of body temperature (i.e., endotherms) and therefore does not apply to ectotherms, some of which also follow Bergmann's rule (Arnett & Gotelli, 2003). There is likely no universal mechanism underpinning Bergmann's rule, regardless of ecto‐ or endothermy. As a result, other mechanisms have been proposed to explain Bergmann's rule, including the starvation‐resistant hypothesis (Arnett & Gotelli, 2003), the diet‐quality hypothesis (Ho et al., 2010), the enemy hypothesis (Manyak‐Davis et al., 2013), the resource rule (McNab, 2010), seasonality hypothesis (Geist, 1987; Huston & Wolverton, 2011), and the temperature–size rule (Atkinson, 1994) (see Section 4 for detailed explanations of these hypotheses).

Fiddler crabs are excellent animals for examining Bergmann's rule. The distribution of each species can span several degrees of latitude, and their morphology varies greatly within that span (Crane, 1975; Darnell & Darnell, 2018). The fiddler crab, *Minuca*(=*Uca*) *pugnax* (Smith, 1870), lives in salt marshes, which are intertidal grasslands, throughout the east coast of the United States. Historically, *M. pugnax* were distributed from northern Florida to Cape Cod, Massachusetts (Williams, 1984), but, like other species, have now expanded their range northward due to ocean warming (Johnson, 2014; Johnson, 2015). Thus, it is a climate migrant.

We had two goals for this study. First, we examined whether *M. pugnax* follows Bergmann's rule. Finding that *M. pugnax* did follow Bergmann's rule, our second goal was to explore the role of temperature in driving the body size patterns for *M. pugnax*. We focused on two temperature‐related hypotheses because temperature is the primary factor driving body size of invertebrates (Atkinson, 1994). The first hypothesis we tested is the temperature–size rule, which states that there is an inverse relationship between temperature and body size (i.e., individuals are bigger at lower temperatures) (Atkinson, 1994). For invertebrates that follow the temperature–size rule, the response is typically phenotypic, not genetic (Ghosh, Testa, & Shingleton, 2013; Kelly, Rivera, Grosholz, & Ruiz, 2015; Stelzer, 2002). Several mechanisms have been proposed to explain the temperature–size rule, including variation in maternal investment, growth rates, and critical size with temperature (Atkinson, 1994; Ghosh et al., 2013; Stelzer, 2002). We do not explore those mechanisms here. Second, we tested the seasonality hypothesis which predicts that the body size of an animal is determined by the amount of overlap between peak resource production and animal growth (Geist, 1987; Huston & Wolverton, 2011). Thus, individuals are largest where seasonality is greatest because the peak pulse of resources (e.g., primary production) overlaps with the timing of animal growth and reproduction.

## 2 METHODS

**Figure 1:**
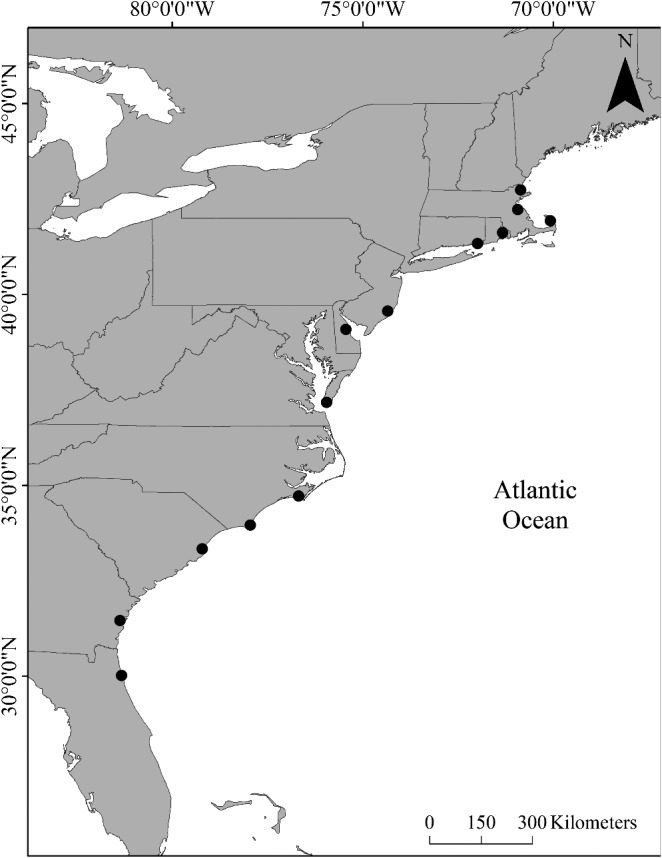
Location of marshes sampled. Figure created in ArcMap (10.5.1)

We sampled 13 marshes that spanned over 12 degrees of latitude, from northeast Florida to northeast Massachusetts, United States (Figure 1, Table 1). Many of these marshes were a part of long‐term monitoring programs such as National Estuarine Research Reserve (NERR) and Long‐Term Ecological Research (LTER) sites (Table 1), which provided temperature data for our analyses (see Appendix 1).

**Table 1:** Location of marshes sampled on the Atlantic coast of the United States with mean (±1‐standard error of the mean) carapace width of male fiddler crabs, *Uca pugnax*

City/town, State	Site	Latitude (North)	Longitude (West)	Carapace width (mm)	
				Mean ± *SEM* (*n*)	Range
Rowley, MA	Plum Island Ecosystem LTER	42°44′20.9″	70°50′56.0″	18.5 ± 0.43 (28)	9.9–22.1
Weymouth, MA	Bare Cove Park	42°13′47.28″	70°55′37.2″	16.2 ± 0.79 (37)	9.0–23.4
Wellfleet, MA	Cape Cod National Sea Shore	41°55′51.24″	70°4′4.8″	16.8 ± 0.40 (27)	10.6–19.9
Prudence Island, RI	Narragansett Bay NERR	41°37′33.24″	71°19′33.6″	17.1 ± 0.42 (29)	13.2–21.9
Noank, CT	Sixpenny Island	41°20′2.04″	71°58′51.6″	14.7 ± 0.41 (33)	11.0–19.5
Little Egg Harbor, NJ	Jacques Cousteau NERR	39°33′56.88″	74°20′31.2″	15.3 ± 0.50 (30)	9.6–21.0
Dover, DE	Delaware Bay NERR	39°5′17.52″	75°26′13.2″	15.6 ± 0.39 (30)	11.5–20.4
Townsend, VA	Virginia Coast Reserve LTER	37°10′26.76″	75°56′31.2″	16.3 ± 0.54 (30)	11.7–21.4
Beaufort, NC	Rachel Carson NERR	34°43′19.56″	76°40′33.6″	12.5 ± 0.47 (25)	9.4–17.6
Kurre Beach, NC	Zeke's Island NERR	33°57′34.56″	77°56′38.4″	12.1 ± 0.34 (30)	8.1–15.7
Georgetown, SC	North Inlet‐Winyah Bay NERR	33°20′6.36″	79°12′18″	13.3 ± 0.44 (30)	8.5–17.3
Meridian, GA	Sapelo Island NERR/ Georgia Coastal Ecosystems LTER	31°27′24.48″	81°21′50.4″	9.9 ± 0.33 (30)	6.6–13.6
St. Augustine, FL	Guana Tolomoto Matanzas NERR	30°1′21.72″	81°19′37.2″	12.4 ± 0.34 (28)	7.6–14.9

Abbreviations: LTER, Long‐Term Ecological Research site; NERR, National Estuarine Research Reserve; *SEM*, Standard error of the mean.

### 2.1 Bergmann's rule

To test the hypothesis that *M. pugnax* follows Bergmann's rule, we collected 25–37 adult male crabs from each marsh from 23 July to 19 August 2016. Crabs were haphazardly collected at low tide in the vegetation of the low marsh, which was dominated by the cordgrass, *Spartina alterniflora*, usually within 5 m of the marsh edge. Crabs were collected by hand from the surface or by coaxing them to the surface by placing a trowel or finger beside their burrows. Crabs were measured for carapace width with digital calipers. Collectors were not aware that crabs would be measured for body size. We recognize that this collection method may still bias our collection toward larger crabs. If this bias exists, we assume that it occurred at all sites. Thus, if mean body size is not accurate for the entire population within a marsh, it is still valid for intermarsh comparisons. Further, in addition to mean body size, we examined the upper decile and maximum body size (see below).

### 2.2 Temperature as a driver of body size

We used mean annual temperature data collected at or near each site to determine its effect on body size. To test the temperature–size rule, we obtained air and water temperature data from monitoring programs (i.e., LTER, NERR sites), nearby weather stations, and ocean buoys for 2016 (see Appendix 1 for sources). Similarly, to test the seasonality hypothesis we used the standard deviation of air and water temperatures as a proxy for seasonality. We tested both air and water temperatures because fiddler crabs are intertidal.

Because our sampling may bias toward collection of larger individuals, we used the following values of carapace width for analysis: mean, mean maximum, and maximum size. Mean maximum was the average carapace width of the largest 10% (i.e., the upper decile) of crabs in each site. Mean maximum is a standard fisheries value used to estimate the upper mean size of a given population (Macpherson & Duarte, 1994; Shin, Rochet, Jennings, Field, & Gislason, 2005). The maximum size is a useful index for estimating the relationship between body size and latitude in case of sampling bias because it looks at the largest individuals within each population (Grosholz & Ruiz, 2003; Kelly et al., 2015).

### 2.3 Statistics

To test the hypothesis that *M. pugnax* follows Bergmann's rule, we conducted simple linear regressions of crab carapace width and latitude for mean, mean maximum, and maximum size separately. All three values were normally distributed based on Shapiro–Wilk's test (*p* > .05), and thus, the data were not transformed prior to analysis. To examine hypotheses related to temperature variables, we conducted multiple linear regressions to test the effect of the following predictors on mean, mean maximum, and maximum *M. pugnax* carapace width: mean annual air temperature, mean annual water temperature, standard deviation of air temperature, and standard deviation of water temperature. Collinear variables, such as water and air temperature, can inflate standard errors (i.e., variance inflation factor, VIF) and increase type II errors. To minimize collinearity, we followed the approach of Zuur, Ieno, and Elphick (2010). We started with the full model (i.e., all variables), calculated the VIFs for each covariate, removed the covariate with highest VIF, and repeated this sequence until all covariates had a VIF ≤ 3. All statistical analyses were conducted in R (version 3.0.3, R Core Team, 2014).

## 3 RESULTS

### 3.1 Bergmann's rule

**Figure 2:**
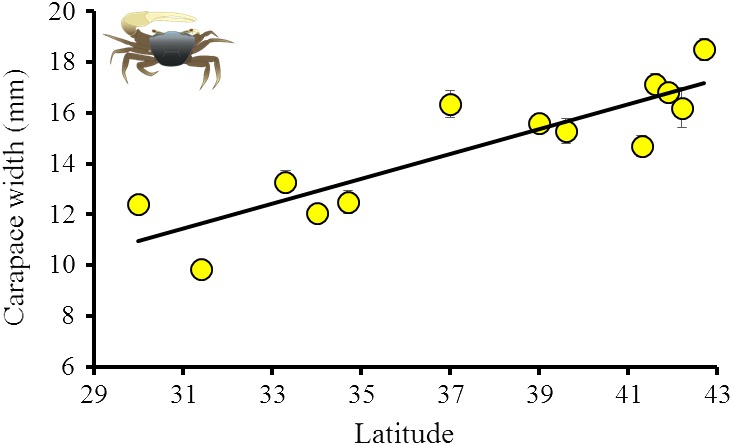
Relationship between latitude and mean carapace width of male fiddler crabs, *Minuca pugnax*. *M. pugnax* image courtesy of Lauren Huey

**Figure 3:**
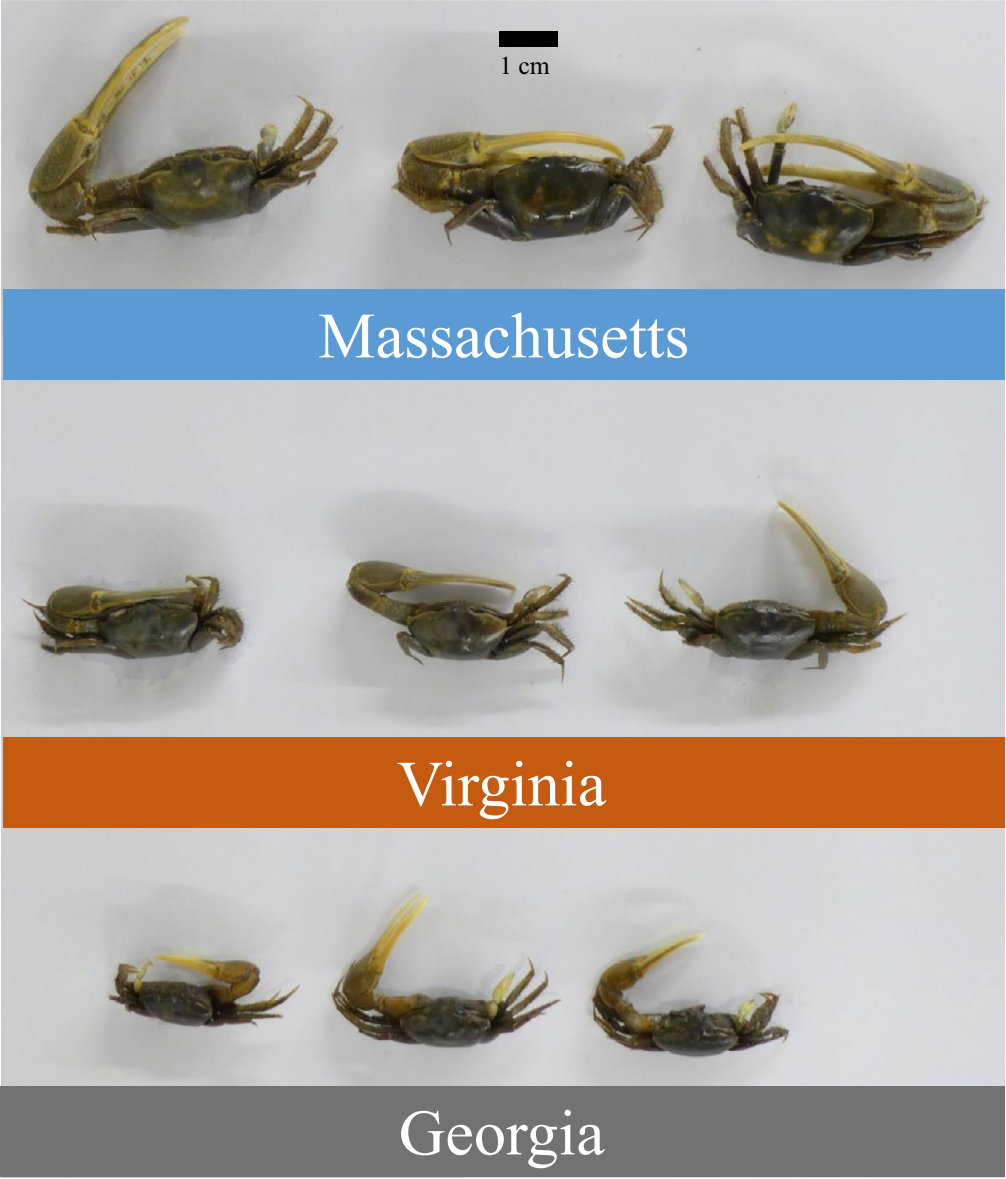
Average‐sized male *Minuca pugnax* from Sapelo Island, Georgia, Cape Charles, Virginia, and Rowley, Massachusetts. Photo credit: DS Johnson

All three measures of *M. pugnax* body size was positively correlated with latitude (linear regression, *p* < .01, Table 2, Figure 2). On average, carapace width increased 0.5 mm for every degree of latitude, suggesting that *M. pugnax* follows Bergmann's rule. On average, the largest crabs were found in the expanded range (northeast Massachusetts, 18.5 mm mean carapace width) and the smallest in the historical range (Georgia, 9.9 mm mean carapace width) (Table 1, Figures 2 and 3).

**Table 2:** Simple linear regressions of latitude and *Uca pugnax* body size variables

Size variable	Estimate	*SEM*	*F*‐value	Degrees of freedom	Adjusted *R* ^2^	*p*‐value
Mean	0.49	0.08	36.8	1, 11	.75	**<<.01**
Mean Max	0.49	0.11	18.3	1, 11	.59	**.001**
Max	0.61	0.09	44.2	1, 11	.78	**<<.01**

Note

Bold indicates significance at *α* = .05.

### 3.2 Temperature as a driver of body size

After sequentially eliminating covariates with VIFs > 3 from the full multiple linear regression models, mean water temperature and the standard deviation of water temperature remained in the reduced model for mean, mean maximum, and maximum body size. These models were significant for all body size variables (*p* ≤ .03, Table 3). Mean water temperature was the only significant predictor of mean *M. pugnax* body size, which was inversely related to body size (*p* < .01, Table 3).

**Table 3:** Reduced models of multiple regressions for *Uca pugnax* body size variables against mean and standard deviation (*SD*) of water temperature

Size variable	Predictor variable	Estimate	*SEM*	*t*‐value/*F*‐value	Adjusted *R* ^2^	*p*‐value
Mean				8.6	.56	**.007**
	Mean	−0.55	0.13	−4.1		**<.01**
	*SD*	−0.12	0.40	−0.3		.76
Mean Maximum				4.9	.39	**.03**
	Mean	−0.53	0.17	−3.1		**<.01**
	*SD*	0.10	0.52	0.2		.86
Maximum				12.3	.65	**.002**
	Mean	−0.70	0.14	−4.8		**<<.01**
	*SD*	0.25	0.43	0.6		.59

Note

Bold indicates significance at *α* = .05.

## 4 DISCUSSION

We found that the fiddler crab, *Minuca pugnax*, like many other marine invertebrates, follows Bergmann's rule (Darnell & Darnell, 2018; Ho et al., 2010; Manyak‐Davis et al., 2013). This was true for its mean, mean maximum, and maximum body size. On average, carapace width increased by 0.5 mm for every degree of latitude increase. Mean water temperature was the strongest predictor of *M. pugnax* body size and was inversely related. Thus, *M. pugnax*, like other ecotherms, also follows the temperature–size rule (Atkinson, 1994). Similarly, Jaramillo et al. (2017) found that the body size of intertidal amphipods, isopods, and crabs was inversely related to sea‐surface temperature (air temperature was not examined).

How do cooler temperatures lead to larger *M. pugnax*? The answer may lie in the time it takes for crustaceans to reach maturity, which relies on molt frequency. The molt frequency of crustaceans declines in colder water, which results in a longer intermolt period, a time of energy acquisition, and, in turn, leads to greater somatic growth at each molt increment compared with individuals in warmer waters (Cunningham & Darnell, 2015; Groner, Shields, Landers, Swenarton, & Hoenig, 2018; Kuhn & Darnell, 2019; Poleck & Denys, 1982). Thus, a cold‐water crustacean will reach maturity at a larger size than their warm‐water conspecifics. Once crustaceans reach maturity, energy is diverted away from growth and put into reproduction (Groner et al., 2018; Landers, Keser, & Saila, 2001; Waddy & Aiken, 1990), thus limiting growth rates and maintaining the size differences for crustacean species in warmer versus colder waters. For instance, American lobsters, *Homarus americanus*, mature at a smaller size in the southern part of their range due to warmer temperatures. When mature, they have slower growth rates because more energy is invested in reproductive rather than somatic growth (Landers et al., 2001; Waddy & Aiken, 1990). An alternative hypothesis is that metabolic constraints underpin the temperature–size rule. Lonsdale and Levinton (1985) suggest that body size is determined by the allometric relationships among body size, food consumption, maintenance, and the temperature at which they are physiologically accustomed. Thus, at warmer temperatures, organismal maintenance (metabolism) may outpace energy acquisition (ingestion) resulting in a smaller body size than at cooler temperatures.

### 4.1 Unexplored drivers of body size

Not all species, even those that follow Bergmann's rule, follow the temperature–size rule. For instance, in the seagrass beds of the eastern United States, the marine isopod, *Idotea balthica*, follows Bergmann's rule (Manyak‐Davis et al., 2013), but not the temperature–size rule. Diatoms shrink or do not respond to higher temperatures (Adams et al., 2013; O'Gorman et al., 2017). Even fiddler crabs have different body size responses to latitude and temperature. For instance, based on air temperature, *M. pugilator*, which co‐occurs with *M. pugnax* on the east coast of the United States, follows Bergmann's rule and the temperature–size rule; however, *Leptuca* (=*Uca*) *panacea*, on the Gulf coast of the United States, does not (Darnell & Darnell, 2018). These results suggest that other factors are either interacting with temperature or independently driving body size in these organisms.

For our study, it is important to keep in mind that our results are correlative based on a biogeographic pattern. Field or laboratory experiments are required to explicitly test mechanisms (e.g., Manyak‐Davis et al., 2013). Additionally, while we find support for the temperature–size rule to explain the body size pattern of *M. pugnax* (i.e., Bergmann's rule), we cannot exclude other unexplored hypotheses. For instance, the presence/absence of enemies such as predators and parasites can influence organismal body size (enemy hypothesis; Manyak‐Davis et al., 2013; Torchin, Lafferty, & Kuris, 2001). Because enemy pressure is related to biodiversity, and biodiversity generally decreases with latitude, enemy pressure also decreases with latitude (deRivera, Ruiz, Hines, & Jivoff, 2005; Manyak‐Davis et al., 2013). Like our results for *M. pugnax*, the body size of the marine isopod, *Idotea balthica*, which is found in seagrass beds within the range of *M. pugnax*, also increases with latitude (Manyak‐Davis et al., 2013). However, based on experimental work, *I. balthica* does not follow the temperature–size rule and the authors found that predation pressure was likely a primary factor influencing isopod body size. Johnson et al. (in revision) found that parasite diversity and intensity in *M. pugnax* hosts decreases with latitude, which may also influence host body size.

The nutritional quality of prey/plants can influence consumer body size (i.e., the diet‐quality hypothesis; Ho et al., 2010). *Minuca pugnax* eats plant detritus, algae (e.g., diatoms), and fungus (Grimes, Huish, Kerby, & Moran, 1989). Within the range of *M. pugnax*, plant nutritional quality increases with latitude in saltmarsh plants such as *S. alterniflora* (Pennings & Silliman, 2005; Pennings et al., 2007); thus, crabs at higher latitudes may benefit from higher quality detritus. However, algae such as benthic diatoms dominate the diet of *M. pugnax* (Grimes et al., 1989) and there is no evidence of a latitudinal trend in the quality of benthic algae in salt marshes found on the United States east coast.

Finally, overwintering starvation may kill off smaller individuals, which have fewer energy reserves to resist starvation (the starvation‐resistance hypothesis; Arnett & Gotelli, 2003; Kaspari & Vargo, 1995). Brodie et al. (2017), however, found no trend in fat storage in *M. pugnax* from Georgia to Massachusetts based on the hepatopancreas somatic index, a measure of stored energy, and thus, there is no support for this hypothesis for this species.

### 4.2 Consequences of body size

Because the impact an organism has on an ecosystem correlates with its body size (Hall, Koch, Marshall, Taylor, & Tronstad, 2007; Woodward et al., 2005), the latitudinal difference in the body size of *M. pugnax* has consequences for its impact on saltmarsh functioning and species interactions. *Minuca pugnax* consumes benthic algae, fungus, and plant detritus (Crane, 1975; Grimes et al., 1989). Larger crabs in the north may have greater grazing rates on benthic algae, fungus, and detritus than those in the south due to size‐driven metabolic demands. *Minuca pugnax* are ecosystem engineers (sensu Jones, Lawton, & Shachak, 1994) that modify marsh habitats through their burrowing activity and can indirectly influence a suite of saltmarsh functions. *Minuca pugnax* can stimulate aboveground plant biomass (Bertness, 1985), reduce belowground plant biomass (Gittman & Keller, 2013), enhance nutrient cycling and decomposition (Holdredge, Bertness, Herrmann, & Gedan, 2010; Thomas & Blum, 2010), change infaunal densities (DePatra & Levin, 1989), and affect sediment erosion or accumulation (Katz, 1980; Smith & Green, 2015). Thus, larger crabs in the northern marshes may have larger per‐capita impacts on saltmarsh functioning relative to their southern counterparts. The total effect of *M. pugnax*, however, will be a product of its population‐level and per‐capita effects. For instance, the total impact of *M. pugnax* at the most northern site studied here (Rowely, Massachusetts) is likely small because their densities are substantially lower (1–6 m^−2^) (K. S. Martinez‐Soto & D. S. Johnson, unpublished data) than those at lower latitudes (60–120 m^−2^) (Culbertson et al., 2007; Luk & Zajac, 2013).

## 5 CONCLUSIONS

In summary, we found that *M. pugnax* follows Bergmann's rule and water temperature is the strongest driver of this body size pattern. These results have important implications for its biogeography and ecology. First, because our oceans and the atmosphere are warming and the body size of *M. pugnax* is inversely related to temperature, we predict that *M. pugnax* body size at a specific latitude will shrink as the climate continues to warm (Salewski, Hochachka, & Fiedler, 2010). Second, as *M. pugnax* expands its range north due to ocean warming in the Gulf of Maine (Pershing et al., 2015), crabs at the highest latitudes will continue to be the largest. Finally, because *M. pugnax* is an ecosystem engineer that influences many saltmarsh functions such as primary production and soil strength (Bertness, 1985; Katz, 1980; Smith & Green, 2015), the body size gradient seen here may influence *M. pugnax's* relative impact on salt marshes throughout its range (Woodward et al., 2005).

## CONFLICT OF INTEREST

None declared.

## AUTHOR CONTRIBUTIONS

DSJ designed the study, analyzed the data, and wrote the first draft of the manuscript. CC, JN, KL, BW, and SW collected samples, generated data and figures, interpreted data, and revised drafts.

## Data Availability

All data are publicly available at the Plum Island Ecosystem Long‐Term Ecological Research website, https://pie-lter.ecosystems.mbl.edu/content/fiddler-crab-body-size-salt-marshes-floridamassachusetts-usa-pie-and-vcr-lter-and-noaa-nerr.

## Appendix 1 Sources for temperature data

**Table:**

Site	Variable	Source
Plum Island Ecosystems LTER	Air temperature	LTER data, http://pie-lter.ecosystems.mbl.edu/content/mon-pr-met15min2016csv
	Water temperature	LTER data, http://pie-lter.ecosystems.mbl.edu/content/mon-so-ibycysi2016csv
Bare Cove Park	Air temperature	National Weather Service for Hingham, MA, http://w2.weather.gov/climate
	Water temperature	NOAA Buoy, Station 44013, Boston, Massachusetts
Cape Cod National Sea Shore	Air temperature	National Weather Service for Chatham, MA, http://w2.weather.gov/climate
	Water temperature	NOAA Buoy, Station 44013, Boston, Massachusetts
Narragansett Bay NERR	Air temperature	NERR data, http://cdmo.baruch.sc.edu/
	Water temperature	NERR data, http://cdmo.baruch.sc.edu/
Sixpenny Island	Air temperature	National Weather Service for Groton, CT, http://w2.weather.gov/climate
	Water temperature	NOAA Station NLNC3, 8461490, New London, CT
Jacques Cousteau NERR	Air temperature	NERR data, http://cdmo.baruch.sc.edu/
	Water temperature	NERR data, http://cdmo.baruch.sc.edu/
Delaware Bay NERR	Air temperature	NERR data, http://cdmo.baruch.sc.edu/
	Water temperature	NERR data, http://cdmo.baruch.sc.edu/
Virginia Coast Reserve LTER	Air temperature	LTER data, Porter, Krovetz, Nuttle, & Spitler, 2017
	Water temperature	Porter, Krovetz, Spitler, Williams, Overman, 2017
Rachel Carson NERR	Air temperature	NERR data, http://cdmo.baruch.sc.edu/
	Water temperature	NERR data, http://cdmo.baruch.sc.edu/
Zeke's Island NERR	Air temperature	NERR data, http://cdmo.baruch.sc.edu/
	Water temperature	NERR data, http://cdmo.baruch.sc.edu/
North Inlet‐Winyah Bay NERR	Air temperature	NERR data, http://cdmo.baruch.sc.edu/
	Water temperature	NERR data, http://cdmo.baruch.sc.edu/
Sapelo Island NERR/ Georgia Coastal Ecosystem LTER	Air temperature	LTER data
	Water temperature	LTER data
Guana Tolomoto Matanzas NERR	Air temperature	NERR data, http://cdmo.baruch.sc.edu/
	Water temperature	NERR data, http://cdmo.baruch.sc.edu/

Note

Water temperature is surface temperature (<1 m).

Abbreviations: LTER, Long‐Term Ecological Research site; NERR, National Estuarine Research Reserve; NOAA, National Oceanic and Atmospheric Administration.
